# Perspectives of Molecularly Imprinted Polymer-Based Drug Delivery Systems in Cancer Therapy

**DOI:** 10.3390/polym11122085

**Published:** 2019-12-13

**Authors:** Andreea Elena Bodoki, Bogdan-Cezar Iacob, Ede Bodoki

**Affiliations:** 1 Inorganic Chemistry Dept., Faculty of Pharmacy, “Iuliu Haţieganu” University of Medicine and Pharmacy, 8 Ion Creangă St., 400010 Cluj-Napoca, Romania; abota@umfcluj.ro; 2 Analytical Chemistry Dept., Faculty of Pharmacy, “Iuliu Haţieganu” University of Medicine and Pharmacy, 4 Pasteur St., 400349 Cluj-Napoca, Romania; iacob.cezar@umfcluj.ro

**Keywords:** molecularly imprinted polymers, chemotherapeutics, cancer therapy, drug delivery systems

## Abstract

Despite the considerable effort made in the past decades, multiple aspects of cancer management remain a challenge for the scientific community. The severe toxicity and poor bioavailability of conventional chemotherapeutics, and the multidrug resistance have turned the attention of researchers towards the quest of drug carriers engineered to offer an efficient, localized, temporized, and doze-controlled delivery of antitumor agents of proven clinical value. Molecular imprinting of chemotherapeutics is very appealing in the design of drug delivery systems since the specific and selective binding sites created within the polymeric matrix turn these complex structures into value-added carriers with tunable features, notably high loading capacity, and a good control of payload release. Our work aims to summarize the present state-of-the art of molecularly imprinted polymer-based drug delivery systems developed for anticancer therapy, with emphasis on the particularities of the chemotherapeutics’ release and with a critical assessment of the current challenges and future perspectives of these unique drug carriers.

## 1. Therapeutic Approaches in Cancer Therapy

Despite the substantial development in early detection and treatment of cancer, malignancies continue to represent the second worldwide cause of death, outranked only by cardiovascular diseases. Malignant processes are triggered by the accumulation of genetic errors that transform normal cells into abnormal ones with limitless and uncontrolled division, and the ability to evade apoptosis and to invade distant tissues [1]. The mutations that initiate a tumor affect both oncogenes that code processes involved in cell proliferation and differentiation, and tumor suppressor genes that code proteins involved in the inhibition of cell growth and the initiation of apoptosis [2].

**Figure 1:**
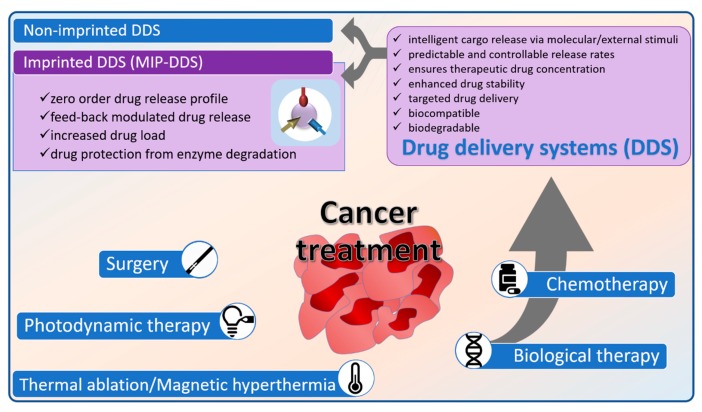
Molecularly imprinted polymeric drug delivery system (MIP-DDS) as controlled and localized drug release systems in cancer therapy.

Surgery and radiotherapy are the elective approaches for local, non-metastatic cancers, while conventional chemotherapy and biological therapies are the efficient alternatives for metastatic tumors (Figure 1). Efforts have been made to combine chemotherapy with radiotherapy and photodynamic therapy (PDT) in the attempt to induce a synergistic antitumor effect and to reduce the dose of chemotherapeutic drugs [3]. The history of chemotherapy spans over almost nine decades and starts with the use of nitrogen mustard and antifolates. A vast and complex body of knowledge is now available but the principles and limitations of chemotherapy revealed by the early research results (1950–1980) still apply. Conventional chemotherapy relies on the inhibition of the replicative potential of malignant cell but its major deficiency is non-specificity. The indiscriminate destruction of both normal and abnormal cells, the severe toxicity and poor bioavailability of conventional drugs, and the multidrug resistance are issues that still need to be addressed by the scientific community.

The novel targeted therapies are able to initiate the discriminatory death of abnormal cells by apoptosis or stimulation of the immune system (a direct approach), or by specific delivery of the chemotherapeutic to cancer cells (an indirect approach) [2,4,5]. The first approach relies mainly on the molecular and genetic bases of the signaling networks that control cell regulation and survival [5]. Growth factors, signaling molecules, cell-cycle proteins, modulators of apoptosis, and molecules that promote angiogenesis, have been identified as potential targets for the new generation of chemotherapeutics [4].

The indirect approach is consistent with the paradigm shift that has been evident during the last decades in the way we address the treatment of chronic diseases. Thus, the focus tends to drop on the efficient delivery of drugs with proven clinical value, rather than on the search for new therapeutic agents. This is particularly valid for cancer treatment in which case high doses of anticancer drugs with non-specific toxicity and poor pharmacokinetics are required [6]. In the case of most traditional pharmaceutical formulations, the severe fluctuations of the anticancer drug’s plasmatic concentration upon systemic administration, lead to high toxicity, poor specificity, and severe side effects. Moreover, the indiscriminate toxicity to normal and cancer cells due to the non-specific drug distribution in the body limits the administrated doses, which in turn may lead to negligible effects on the ultimate target. In order to achieve maximum therapeutic effects at a specific target and with minimum adverse effects, the aforementioned problems associated with the conventional pharmaceutical formulations must be addressed as they are listed among the main causes of the drastic decrease of the therapeutic value of many anticancer drugs [7].

The loading of anticancer drugs within different drug delivery systems (DDS) plays a significant role in improving treatment efficiency through multiple ways, mainly by an improvement of the pharmacokinetic and the pharmacodynamic profile of the chemotherapeutic. An efficient DDS should ensure the controlled accumulation of the payload within tumors while avoiding normal tissues. Ideally, DDS must be capable of intelligently releasing their cargo as a response to the local environment, at predictable rates, and of maintaining the drug concentration for the required amount of time [8]. Additionally, these carriers may also provide means to improve drug solubility (i.e., curcumin (CUR), capecitabine (CAP) and to protect the payload against premature degradation (i.e., irinotecan). Additionally last, but not least, the carrier should be biocompatible and biodegradable.

It is also noteworthy that while the use of DDS is improving the therapeutic index and safety profile of chemotherapeutics, it also exerts a beneficial effect on the patient’s compliance to treatment. Thus, by decreasing dosing frequency and using patient-friendly delivery devices, self-administration is promoted [9].

The progress in drug delivery technology and the successful clinical translation of earlier macro- and micro-drug delivery systems in the last few decades, has led to the emergence of nanodelivery platforms [10]. The most common nanocarriers for controlled drug delivery applications usually imply inorganic, organic, and hybrid materials. Polymeric nanocarriers, in particular, stand out due to their versatility and highly tunable features allowing targeted and sustained drug release tailored for different administration routes and pathologies. The payload (chemotherapeutics) may be either enclosed within the core region of a core-shell polymeric matrix (e.g., polymeric micelles and nanocapsules), or wrapped on the surface or dispersed within a polymeric network (e.g., nanospheres).

A step further in the design of polymeric nanocarriers represents the loading of the chemotherapeutics through molecular imprinting, a technique that enables the creation within the polymeric matrix of active sites complementary in size and functionality with the drug template. These highly specific sites are not only able to increase the loading capacity of the polymeric nanocarrier, but add an extra boost to the tunability of the drug release kinetics. Furthermore, by careful design, a physical or chemical stimulus-responsiveness of the resulting molecularly imprinted polymeric drug delivery system (MIP-DDS) may also be introduced, offering attractive means of controlling the localized release of the chemotherapeutics (Figure 1).

Despite the advances made in the synthesis of MIPs intended for drug delivery and the numerous studies that offer substantial proof-of-concept, the development of MIP-DDS for cancer therapy is still in its infancy. More work is required to decode their full potential, to overcome some of the issues related to the imprinting process currently not in line with the modern pharmacotherapeutic requirements [11], to assess their safety profile, and finally to reach the ultimate goal, clinical translation.

Excellent reviews on MIP-DDS intended for various routes of administration (topical, enteral, and parenteral) have been published by Lulinski et al. and Tuwahatu et al. [12,13,14,15]. Nevertheless, our review aims to summarize the present state-of-the art of MIP-based DDS focused on anticancer therapy, emphasizing particularities of the chemotherapeutics release and discussing the current challenges and future perspectives of these unique drug carriers. A critical evaluation is performed on why the current research exploiting this particular application of these smart materials delivering chemotherapeutics seems to be stuck in early stages of design and why their translation into preclinical/clinical studies is lingering.

## 2. Non-Imprinted DDS for Cancer Treatment

Without a doubt, a key challenge in cancer therapy is to deliver chemotherapeutics selectively to the tumor site while minimizing their accumulation to healthy tissues. Such goal is difficult to be achieved, especially for small molecular drugs. One approach could be nanocarrier-based delivery that can ensure the successful translation of chemotherapeutic within tumor tissues via the EPR effect (enhanced permeability and retention effect). On a second note, although the literature addressing the fate and effects of nanomaterials on health and the environment is becoming more robust, there are still some key knowledge gaps in terms of their safety profile that remain almost completely unaddressed, especially for systemic nanodelivery systems.

Indeed, nanodelivery of chemotherapeutics is a thriving field, its undeniable potential being reflected by the number of nanocarrier-based drugs that have entered different stages of clinical trials in the past decades [16]. To date however, the number of nanocarrier-based drugs approved for the treatment of cancer is scarce and they are mainly based on liposomes and polymer-drug conjugates [2,17,18,19]. Biocompatible and biodegradable, and with a bilayer structure analogous to that of cell membrane, liposomes are among the most studied DDSs used for the delivery of both hydrophilic and hydrophobic compounds. Liposomal formulations of daunorubicin (DaunoXome^®^), cytarabine (DepoCyt^©^), vincristine (Marquibo^®^), irinotecan (Onivide^®^), doxorubicin (Doxil^®^/Caelyx™), all improved the delivery of the bioactive to tumor sites and therefore lowered the systemic toxicity of the loaded chemotherapeutic. Vyxeos^®^, a liposomal combination of cytarabine and daunorubicin, approved by the FDA in 2017 for the treatment of acute myeloid leukemia, ensures the co-loading of two molecules with synergistic antitumor activity and the sustained release of the payload. The reticuloendothelial system, opsonization phenomena, immunogenicity, and the tendency to accumulate to organs such as the spleen and the liver which can lead to a delay in the removal of the anticancer drugs, are issues commonly related with the use of conventional liposomes as DDS [20]. Different types of liposomes, such as the PEGylated structures (stealth liposomes) approved for the delivery of irinotecan (Onivide^®^) and doxorubicin (Doxil^®^/Caelyx™), were developed in the attempted to overcome the aforementioned problems.

Extensively reviewed [2,10,21,22,23,24], conventional, non-imprinted polymeric nanoparticles (NPs) are known to provide a controlled release of the chemotherapeutic of both hydrophilic and hydrophobic nature and can be engineered to exhibit a responsive behavior. Materials currently used for NP synthesis are biodegradable and biocompatible synthetic or natural polymers that already have FDA approval. With respect to their natural counterparts, synthetic polymers such as polyglycolic acid (PGA), polyethylene glycol (PEG), polylactic acid (PLA), polylactic-co-glycolic acid (PLGA), or N-(2-hydroxypropyl)-methacrylamide copolymer (HPMA) offer the advantage of a long-term sustained release of the payload [19]. The main drawbacks in the case of natural polymers such as albumin, chitosan, alginate, dextran, or collagen are their relatively fast release profile and the problems related to purity and homogeneity [25].

Polymer-drug conjugates have already been approved for the delivery of several anticancer drugs [26]. The leuprolide acetate-PLGH (poly(DL-lactide-co-glicolide) (Eligard^®^) conjugate has an increased circulation time and ensures the controlled delivery of the payload. For the PEG-protein conjugates, i.e., the PEGylated L-asparaginase (Oncaspar^®^) and the PEGylated granulocyte colony stimulating (GCSF) protein, PEGylation improves the stability of the protein of interest in the therapy of cancer [20].

## 3. Molecular Imprinting

Molecular imprinting technique allows the creation of polymeric networks bearing specific binding sites for a predetermined molecule (drug template) via polymerization [27,28,29,30,31]. MIP synthesis is a relatively straightforward and inexpensive procedure, based on mixing the template molecules with a proper functional monomer, in the presence of a crosslinking agent and an initiator in a porogenic solvent. Subsequently, the polymerization is initiated via photo- or thermal-initiation, or an electropolymerization is performed. At the end of the polymerization reaction the exhaustive template removal from the imprinted polymer is a mandatory and critical step in analytical applications, unveiling the imprinted cavities for the subsequent molecular recognition stage. In drug delivery applications however, as long as non-toxic monomers are used, no template extraction is needed. The key to achieving well-defined imprinted cavities relies on choosing the right monomers able to form a template-functional monomer complex that will “lock in place” the template molecule throughout the polymerization process [32], and to provide an optimal rigidity of the polymeric matrix. In analytical applications (separation [33,34,35,36,37] and sensing [38,39,40,41,42]), the MIPs are expectedly highly crosslinked, the rigid networks retaining the sterical and chemical complementarity of the imprinted cavities towards the template, in order to later ensure the specific rebinding of the template molecule. In drug delivery, however, other features that usually require a lower degree of cross-linking, become primordial, e.g., the controlled diffusion of drug template out of the polymer matrix, or the required morphological changes of the polymer (e.g., swelling) in response to external or internal stimuli.

The key quantitative parameter in benchmarking the efficiency of the molecular imprinting process is the imprinting factor (IF), calculated as the ratio of chromatographic capacity factor/relative response (in separation sciences/sensing) or loaded amount of drug (in drug delivery) of the imprinted—compared to non-imprinted polymer. For MIP-DDS the amount of drug load specifically bound into the imprinted sites of the polymer, and ultimately the attained IF, is determined by comparative rebinding studies (MIP versus NIP) upon template removal.

Although the molecular imprinting process for analytical purposes and drug delivery is basically identical, the optimization of MIPs synthesis should be conducted differently, fitted for the distinctive goals of the two applications, i.e., specific molecular rebinding compared to reservoir for controlled drug release. Rooted from these operational differences, the expected features (e.g., cross-linking, swelling degree) of the resulting imprinted polymers are discordantly ranked, implying certain constrains and limitations in the design space or even requiring different imprinting strategies (Table 1).

The imprinting technology has been employed successfully in the development of MIP-based sorbents, stationary phases, sensor interface, or as alternative to biological antibodies and receptor systems [43,44,45,46,47], but the application of MIPs in drug delivery is still in its developing stage, in the in vitro proof of concept phase. However, MIPs possess a great potential for DDS use, due to some unique features compared to conventional polymers. Their particular physicochemical properties allow them to prolong the release profile and to protect the active ingredient (anticancer drug) from enzyme degradation during its transit through the body. Moreover, the tailor-made affinity between the drug template and polymer functional groups introduced by molecular imprinting, endows polymers with a higher drug loading ability compared to the non-imprinted ones. In addition, the already published studies showed that MIPs are capable of releasing the imprinted template in a more sustained way and a zero order drug release could be achieved over long periods of time, this representing a clear advantage over conventional drug delivery [26]. Ideally, MIP-based DDS should be able to release the payload within its therapeutic range, thus reducing the frequency of drug administration and the chemotherapeutics’ side effects. Moreover, drug release can be modulated by a feed-back mechanism in which a specific stimulus (i.e., a change in a biomarker concentration) will initiate unloading of the drug, at a specific site in the human body; the drug release continues for as long as that biomarker is above a certain limit and stops when the biomarker’s level drops. Obviously, as it is also the case for the non-imprinted DDS, other physical (temperature, ultrasound, light, magnetic, or electric field) or chemical (pH, redox potential, ionic strength) stimuli remain viable options to be exploited for the controlled release of the chemotherapeutics from the MIP-based DDS. Not ultimately, the imprinted polymer should be biocompatible, non-immunogenic, and biodegradable.

However, most of the studied MIPs intended for demonstrating their applicability as DDS employ formulations (type and molar ratios of functional monomers, cross-linkers, and porogens) initially tested for analytical applications. No real effort is channeled towards adapting the MIP formulation to the design of drug nanocarriers in line with current pharmaceutical and biomedical regulations, in agreement with minimal safety profile requirements and compliance for medium to long term exposure of the human body (organs, tissues, cellular environment, biological fluids). Moreover, only a small number of studies progressed to in vivo animal model assessment of the MIP-DDS performance and cellular toxicity.

## 4. Molecular Imprinting Approaches in MIP-DDS Development for Cancer Therapy

Although continuous innovations in terms of materials science and conformation design aim to correct most of the shortcomings of conventional NP formulations, they may sometimes fail to significantly improve the activity of anticancer agents due to various reasons, such as poor drug loading capacity (as a result the concentration of drug at the targeted tissue (tumors) is below therapeutic levels, or the important amount of nanocarrier material required leads to undesirable side-effects or toxicity) and/or fast and premature release of the encapsulated drug (leading to a suboptimal activity at the targeted site and an increased number of side effects) [48]. Therefore, there is still room for improvements of DDSs intended for cancer therapy and molecular imprinting might be one viable strategy.

### 4.1. Non-Covalent Imprinting

In non-covalent imprinting, regardless of the intended application (analytical or drug delivery), the imprinting efficiency is strongly influenced by the nature of monomers and molar ratios of template, functional monomer, and cross-linker. The interactions between monomers and template are either covalent or non-covalent (e.g., ionic and hydrogen bonds, hydrophobic interactions, dipole-dipole interactions, van der Waals forces). By far, the most frequently employed approach for the development of MIPs for analytical applications is the non-covalent one as it is applicable to almost any type of template; moreover, the synthesis protocol is much simpler than the one employed in the covalent approach. The non-covalent technique, in principle, can be used to imprint any drug regardless of its chemical structure. Moreover, the weak non-covalent template—monomer interactions ensure the preservation of the therapeutic effect of the antitumoral agents, as Yokohama et al. [49] proved using adriamycin-loaded polyaspartate-PEG micelles. When adriamycin was covalently entrapped via amide bonds in the micelles, the loaded polymeric micelles showed negligible in vivo antitumor activity. Therefore, the main challenge is to obtain the maximum imprinting efficiency able to prevent premature release, while allowing in vivo time-controlled environmentally responsive drug delivery.

The vast majority of the developed imprinted DDS for cancer treatment applications is based on the use of the same acrylic monomers as in the case of MIPs designed for sensing and separation applications [43,50]. It can be noticed that, most often, the formulation of the polymerization mixture was adopted for drug delivery with no or minimal optimization thereof. Since its carboxyl group can develop weak electrostatic interactions and/or can act as a hydrogen donor for N, O, or S containing drugs, methacrylic acid (MAA) remains the most frequently used functional monomer in the non-covalent molecular imprinting protocols for DDS development. Several anticancer drugs were successfully imprinted using MAA, i.e., 5-fluorouracil (5-FU) [51,52,53], CAP [54], paclitaxel (PCX) [55,56,57,58,59], thalidomide [60,61], mitoxantrone [62], and sunitinib (SUT) [63]. Moreover, as it can be inferred from Table 2, the bulk of protocols employed ethylene glycol dimethacrylate (EDMA), an acrylic crosslinker characterized by low biocompatibility and no biodegradability, and therefore with limited perspectives in drug delivery development [64].

As an example, no specific design or particular adaptation of a previously tested MAA/EDMA-based polymerization mixture for α-tocopherol extraction [65] is performed while developing SUT—imprinted DDS (molar ratio of 0.5:16:25 SUT/MAA/EDMA) [63]. Both SUT-MIP and NIP presented a “burst” release of the loaded drug of around 50% within 1 h, reaching after 6 h, 58% for the MIP, and 90% for the NIP, respectively. After 24 h, the SUT-imprinted polymer released 76% of its drug load, indeed demonstrating a retarded release of the template. However, the molecular imprinting efficiency for the synthesized MIP was merely 1.52.

It is also true, that attempts in improving DDS biocompatibility may come with a price to pay in terms of drug loading performance. The ionizable MAA, and 2-hydroxyethyl methacrylate (HEMA), a more biocompatible, non-ionic monomer, were comparatively tested for the synthesis of 5-FU-imprinted particles using precipitation polymerization in acetonitrile (ACN) [51]. A slightly lower imprinting efficiency occurred with HEMA (IF = 2.8) as compared to MAA (IF = 4), accounting for the higher drug release fractions of HEMA-based MIPs, probably due to the weaker bonding of 5-FU with HEMA as compared to MAA.

Using mixtures of these monomers may also have unforeseen outcomes, advocating the need of careful optimization of the MIP-DDS synthesis. The highest drug loading capacity for the 5-FU-imprinted MAA, HEMA, and EDMA-based hydrogel was achieved for an intermediate molar ratio of MAA:5-FU (8:1). At lower 5-FU concentration (16:1 MAA:5-FU molar ratio), the large excess of carboxylic functional groups (MAA) randomly distributed throughout the polymeric network leads to a lower affinity towards the template. In return, in case of higher amounts of template (4:1 MAA:5-FU molar ratio), the scarcity of MAA functional groups became a limiting factor [52].

Another experimental variable with a great impact on the MIPs performance is the type of cross-linker. Schroeder et al. [55] investigated the influence of the crosslinker’s nature on the resulting MIPs for drug delivery purposes. Two different crosslinkers, namely EDMA and trimethylolpropane trimethacrylate (TRIM), were used to synthesize PCX—imprinted MIP microparticles, and their physicochemical and adsorption properties towards PCX, as well as their in vitro activity towards various cancer cell lines and normal human cell lines were tested. Even though drug release lasted for around 50 h in both cases before reaching a plateau, the overall cumulative drug release (85% of total PCX) and its PCX rebinding properties were much higher in the case of TRIM-based MIP as compared to EDMA-based MIP (40% of total PCX). The observed difference was accounted for by the higher cross-linking ratio and bolstered stiffness of the polymeric network induced by tri-functional crosslinker TRIM in comparison with its bi-functional counterpart, EDMA.

Apart of tuning the release profile of the chemotherapeutics, the advantage of using crosslinkers with higher functionality (TRIM versus EDMA) may have beneficial effects in terms of improved drug loading capacity and selectivity (rebinding efficiency of MIP versus NIP), as demonstrated by Ishkuh et al. [59] by the synthesis of PCX-imprinted NPs using mini-emulsion polymerization. The carboxylic groups of MAA were used as hydrogen bond donors and acceptors to mediate specific interactions between the polymeric network and the functional groups of PTX. In addition to the impact of the crosslinker’s nature on the resulting MIP-DDS performance, it was found that higher molar ratios of the crosslinker are beneficial for the imprinting process. A molar ratio of 0.25:3:8 PCX:MAA:TRIM was found to be optimal as it showed the best imprinting efficiency for PCX. The imprinted NPs prepared with TRIM showed improved drug loading capacity (17.8%), with a 12 times higher binding efficiency compared to NIP NPs in biological samples. During 28 h, TRIM-based MIP released 14.4% of PCX, followed by a decrease in the release rate, reaching only around 20% of the loaded-drug in two weeks.

Although attempts in developing formulations with multi-analyte(drug) imprinting capability [66], currently there are still no universally applicable, rational guidelines in selecting the ideal composition of the polymerization mixture, and MIP-DDS optimization is still very much relying on an empirical approach. Obviously, not only qualitative variables (nature of functional monomer, cross-linker, porogen) have to be tailored to the chemical structure of the chemotherapeutics and type of imprinting approach, but also the molar ratio of each individual component of the polymerization mixture. The careful optimization of these quantitative variables may have a critical role in the resulting MIP-DDS’s drug loading capacity (imprinting factor; selectivity of template rebinding—homogeneity of imprinted sites) and drug release profile (through the chemical (degree of cross-linking, hydrolytic/enzymatic cleavage) and the mechanical (stiffness, swelling capacity) properties of the polymeric matrix). As such, during the preparation of 5-FU-imprinted NPs by precipitation polymerization in a mixture of 1:1 methanol:ACN as porogenic solvent, besides studying the impact of two chemically related monomers, namely acrylamide (AM) and N,N′-methylenebis(acrylamide) (MBA), the effect of template-functional monomer ratio on the drug release and rebinding profile was also monitored [67]. Interestingly, the rebinding efficiency (up to 84.53%) in the case of the AM-based MIPs increased with the decrease of 5-FU molar ratio used during synthesis; the release rate presented, however, a contrary trend. For the MIPs prepared with MBA as monomer, rebinding efficiency was positively correlated with the tested template ratios, however their release profiles were very similar in all cases. AM as functional monomer and EDMA as crosslinker were also employed in the synthesis of doxorubicin (DOX)-imprinted DDS [68]. Even though the imprinting was based on the hydrogen bonds between AM and template, the polymerization was conducted in a polar solvent, namely ethanol, and the DOX:AM:EDMA ratio was 1:25:112.5. The dissociation constant (K_d_) and the maximum binding number (Q_max_) were 35.6 μmol·g^−1^ and 3.4 μM for the MIP, and 12.5 μmol·g^−1^ and 100 μM for the NIP.

The ideal template to functional monomer/crosslinker pairing (azidothymidine (AZT):itaconic acid (ITC):EDMA = 1:2:20), combined with the appropriate type of molecular imprinting/polymerization (bulk/surface imprinting, bulk/precipitation polymerization) has been empirically demonstrated on the imprinting/rebinding efficiency and in vitro cell cytotoxicity of AZT-loaded DDS [69]. ITC proved to be the best functional monomer for AZT imprinting, probably because of the two carboxylic acid functionalities; a higher IF (1.47) was achieved for ITC as compared to MAA (IF = 1.03). Polymers crosslinked by EDMA presented a superior IF (1.86) as compared to trimethylolpropane triacrylate (TMPTA) (IF = 1.013), even though EDMA has only two functional vinyl groups, and in general trifunctional crosslinkers are considered superior in terms of imprinting efficiency and loading capacity [70]. As expected, the precipitation polymerization method offered higher IF and rebinding capacity as compared to traditional bulk polymerization, due to the formation of uniform spherical polymeric particles with a narrower size distribution. Moreover, the superiority of surface imprinting on vinyl-modified silica-coated magnetic (Fe_3_O_4_) NPs (IF = 4.57) over the conventional bulk imprinting approach, has also been demonstrated. The highest rebinding capacity, via a Freundlich mechanism and fast pseudo second-order kinetic adsorption (5 min), was provided also by the magnetic MIP: 45.83, 18.16, 170.75, and 37.74 mg/g for MIP, NIP, magnetic MIP, and magnetic NIP, respectively. The recorded in vitro cell cytotoxicity of magnetic MIP, MIP, and free AZT on MCF-7 cancer cell lines, was 91%, 71%, and 11%, respectively, with no harmful effect on the normal cell line observed.

To simplify the empirical design space in paring the appropriate functional monomer to the drug template, thermodynamic computational calculations may be employed for the prescreening of functional monomer candidates likely to form stable complexes with the drug molecule in the pre-polymerization step. As such, 4-vinylpyridine (4-VPy) and acrylic acid (AA) were selected as optimal functional monomers for the preparation of MIPs for the controlled release of 5-FU [71]. The polymerization was performed in a mixture of ACN/methanol 80:20 (v/v), using EDMA as crosslinker. It was found that the release rate for magnetic MIP containing 4-VPy was higher than in the case of the AA-based MIP, but not to a significant extent.

**Figure 2:**
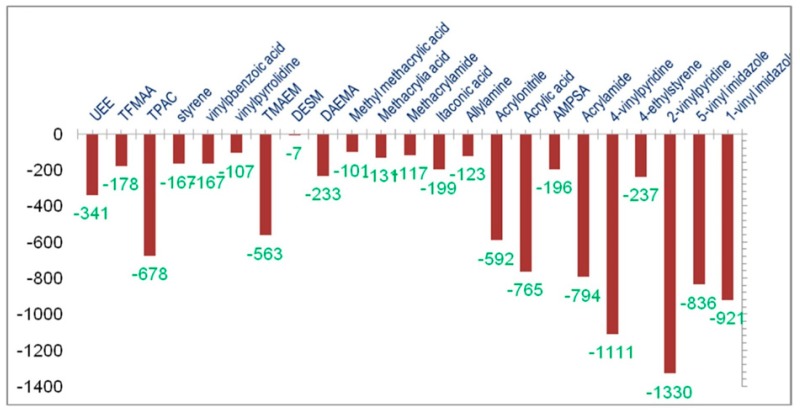
Combinatorically screened functional monomers for the design of amygdalin nanoMIP. Reproduced with permission from [72].

In a similar manner, 4-VPy and 2-VPy were selected as the functional monomers with the highest affinity for amygdalin, based on the computationally determined binding scores from a virtual library of 23 functional monomers (Figure 2) [72]. The molecular complex between 4-VPy and amygdalin presented the highest binding energy of −1330 kcal·mol^−1^, whereas 2-VPy and template showed a binding energy of −1111 kcal·mol^−1^.

From such combinatorial approaches one may try to establish the nature of binding interactions (i.e., H-bonding), the involved functionalities (i.e., hydroxyl group of amygdalin and the nitrogen atom of VPy), and also to estimate the influence of the employed crosslinker on the stability of pre-polymerization complex, which in the case of EDMA, was considered negligible (0.04 kcal·mol^−1^ contribution to the binding energy). Nevertheless, the overall impact of the crosslinker on the MIP-DDS’s drug release profile, or the influence of various molar ratios of polymerization mixture constituents may not be accounted for by this approach. The up to 70% release of amygdalin from the imprinted polymers followed a Fickian behavior, while the subsequent drug release follows a zero-order kinetics. An initial burst release could be observed for the first several hours, and 100% drug release was reached within seven days. Experimental data showed an improvement of drug loading capacity by using higher molar ratios of template during MIP preparation.

The morphology, physico-chemical properties, and pharmacokinetic performances of the imprinted polymeric materials are greatly affected by the polymerization mixture constituents [73]. As already mentioned, one pre-requisite of attaining a high imprinting efficiency is a strong binding interaction of the drug template-functional monomer(s) complex. In one study [54], the complex optimization was performed using molecular modeling, by calculating the binding energy between the template CAP and the functional monomer (MAA), at different template:monomer ratios. The corresponding calculated binding energies for 1:4, 1:5, and 1:6 CAP:MAA ratios, were −31.64, −57.06, and −67.27 kcal·mol^−1^, respectively. However, after the MIP preparation and rebinding assessment, the highest IF (4.6) was obtained for the 1:5 CAP:MAA ratio, while for a lower template:monomer ratio (1:6), the IF decreased to 1.2, suggesting that other spatial factors may affect the imprinting process. In addition to MAA and EDMA as crosslinker, a series of liquid crystalline (LC) and polyhedral oligomeric silsesquioxanes (POSS) monomers were added to the polymerization mixture, and a two-fold increase of the IF as compared to the conventional MIPs without LC and POSS was achieved. Moreover, in line with the improved IF, the LC-POSS MIPs displayed the highest absolute and relative (%) amount of loaded drug, with the best encapsulation efficiency, showing also the longest release time, up to 13.4 h.

### 4.2. Covalent Imprinting

**Figure 3:**
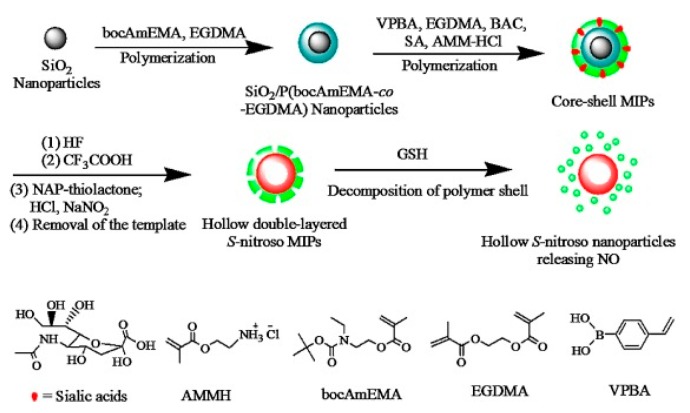
Synthesis protocol of hollow double-layer S-nitrosothiols MIPs. Reproduced with permission from [76].

Although much more popular, the non-covalent approach can be inefficient in drug imprinting for drug delivery applications because of the relatively weak interactions between the template and the functional monomer [74]. It has been shown that a higher yield of specific and more homogeneous binding sites along with reduced non-specific adsorption can be achieved employing reversible covalent bonds between the functional monomer and the template. Such reactions include reversible esterification or condensation reactions, i.e., boronate ester, ketal/acetal, and Schiff base formation. The main disadvantage of this approach is, however, its applicability limited to a relatively low number of template molecules bearing specific functional groups amenable to covalent bond formation or the potential loss of anticancer activity upon covalently binding one of the drug’s functional moiety (i.e., adriamycin [49]). Ideally, the template—monomer bond should have no effect on the drug’s activity, it should be stable in the bloodstream, and should be easily cleaved by either the acidic media or by the lysosomal enzymes within the tumor environment [75]. Addressing these requirements is a serious challenge and it is the main reason why the development of DDS imprinted with chemotherapeutic agents via reversible covalent bonding is limited. To date there is only one study [76] that reports the reversible boronate ester formation in preparing sialic acid (SA)—imprinted hollow double-layer NPs with the aim of targeting tumor cells. These MIP NPs were also imprinted with S-nitrosothiols by grafting on the polymer’s free secondary amines N-acetyl-D-penicillamine thiolactone (NAP) in order to provide thiol groups; the subsequent nitrosylation allows for NO-release in chemotherapy (Figure 3). The resulted DDS was tested in vitro on SA residues over-expressed human cancer cells, and in vivo on HepG2 tumor-bearing BALB/c mice. The results showed an increased bio-distribution of the imprinted DDS, intracellular GSH induced decomposition and rapid NO-release in tumor cells compared to cells without over-expression of SA residues, and also a significant increase of the survival rate in treated mice. Even though a small amount of NO leakage may occur in normal tissues, the authors affirm that the released NO would take part in normal physiological activities and would not cause serious side effects.

### 4.3. Metal Ion-Mediated Imprinting

Conventional imprinting polymerization involves most often the use of aprotic, organic solvents in order to favor the functional monomer-template complex formation during polymerization. Their use however raises safety concerns if the polymer is intended for biomedical applications. An alternative in achieving high affinity imprinted sites in aqueous solutions is the metal ion coordination approach that employs a metal ion as a mediator in the formation of a ternary complex between the functional monomer, the metal ion, and the drug template [44]. The metal pivot interacts with the heteroatoms of the functional monomer and the template by accepting their electrons in order to fulfil its orbitals of the outer coordination sphere. Depending on the nature of the metal ion, its electron configuration, oxidation state, coordination numbers, preferred geometries and ligand preference, a high degree of versatility in tailoring the kinetics and strength of individual interactions can be achieved [77]. The metal-coordination interactions are spatially oriented and stronger than the non-covalent ones, offering good opportunities to design MIPs in protic solvent environment, water in particular, and additionally this can reduce the random incorporation of template. Moreover, they are susceptible to external stimuli, such as pH, temperature, or external ligands, making them applicable to regulate the drug release ratio [78].

**Figure 4:**
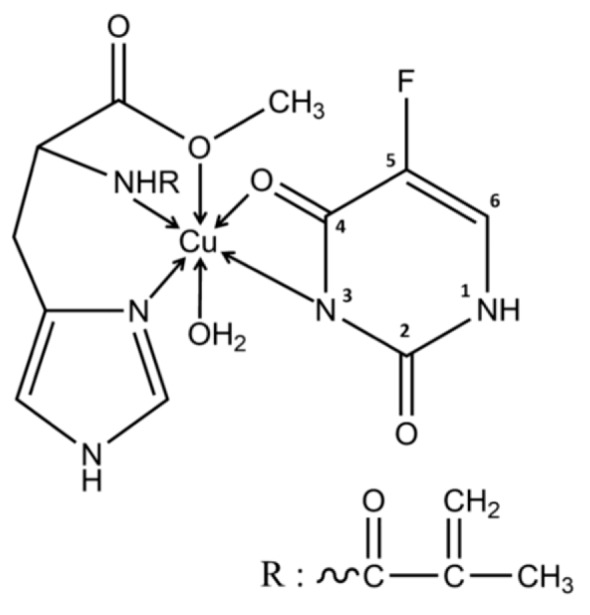
Hypothetical model of the N-methacryloyl-(L)-histidine methyl ester-Cu(II)-5-fluorouracil complex in the preorganization mixture. Reproduced with permission from [78].

The nature of the metal ion is the most important parameter in order to achieve a high imprinting factor, however it needs to satisfy some prerequisites for drug delivery applications; the metal ion should not hamper the polymerization process, it must have a known coordination behavior such as the most favorable template, metal ion, and monomer interactions to be achieved, and most importantly to be non-toxic. The list of metals that have been used in the imprinting process as pivots is quite short, being restricted to several transitional metals such as: Co(II), Co(III), Cu(II), Ni(II), Zn(II), Cd(II), Fe(II), and Fe(III) [44]. Cadmium does not occur naturally in biological systems and is an exceptionally toxic heavy metal [79], thus it cannot be used in DDS development. The other listed metals are essential trace elements and they are generally required as cofactors for enzymatic reactions; in relatively large amounts however, they are harmful or toxic. To the best of our knowledge, the only metal ion used in DDS synthesis designed for cancer treatment is Cu(II) [78,80,81]. The Cu(II) release from the polymeric matrices as a result of the cleavage of coordination bonds could be however the cause of copper-related toxicity. The Recommended Dietary Allowance (RDA) for copper is 900 μg/day for adults and the tolerable upper intake level (UL) is 10,000 μg/day (10 mg/day), the safe upper level of copper intake proposed by the World Health Organization [82]. In all reported studies, the imprinted polymers contained Cu(II) amounts much lower than the UL. In two studies, a polymerizable derivative of L-histidine, namely N-methacryloyl-(L)-histidine methyl ester (MAH), was used as functional monomer and metal-chelating ligand for the interaction with the template, represented by the 5-FU [78] or mitomycin C (MMC) [81] via Cu(II)—mediated coordination. Testing in various receiving media (pH 4.0–7.4) at 37 °C no copper leakage from the imprinted polymers has been detected by graphite furnace atomic absorption spectroscopy, suggesting that Cu(II) ions are strongly chelated to MAH (Figure 4) in tridentate manner through the imidazole ring –N, amino –N, and deprotonated carboxylato –O atoms [78]. It was also reported that Cu (II) could bridge between DOX and 4-VPy, to form a ternary complex consisting of two molecules of DOX, two molecules of 4-VPy, and one Cu(II) ion. The in vitro drug release profiles were influenced by pH, showing a slow release rate at or around physiological pH, whereas in more acidic pH (5.0 or below) a 6-fold increase of the drug release is achieved [80]. The imprinted polymer exhibited an IF of 2.7, with a maximum absorption capacity of 6.74 μmol·g^−1^, the payload being released in a sustained manner within five days. A steeper release profile can be observed in the case of 5-FU [78] and MMC [81] imprinted DDS, nearly 80% of drug being released in just several hours, after reaching the plateau.

## 5. Stimuli Responsive Imprinted DDS for Cancer Treatment

The ideal DDS in cancer therapy should be able to offer a localized, temporized, and dose-controlled delivery of the antitumor agent, dependent on endogenous or exogenous stimuli, via chemical, biochemical, or physical routes [10]. Pathological tumor sites exhibit local biochemical abnormalities, (such as lower pH, elevated reactive oxygen species and enzyme levels, high temperature, overexpressed proteins) which can be used to trigger and activate drug release [83]. Typical exogenous stimuli that are manipulated from outside the body are light (visible, near-infrared, infrared), ultrasound, electric pulses, and magnetic field.

Among the internal stimuli, the change in pH is the most employed strategy for developing MIP-based responsive systems for cancer treatment. The acidic pH in tumor environment is exploited to promote and control drug release. While normal cells have a physiological pH of 7.5, the solid tumors environment is characterized by a pH between 6 and 7, while subcellular compartments, such as endosomal and lysosomal vesicles, a pH below 5.5 [84]. These acidic pH values can be exploited to disrupt the acid-labile bonds of the imprinted delivery system, represented by (i) the template-functional monomer interactions and/or (ii) the covalent bonds from the crosslinked polymer network, leading to an increase of drug release.

The metal ion coordination approach was exploited in the development of pH responsive imprinted polymers for the sustained release of DOX [80] and 5-FU [78], based on the template—Cu(II)-functional monomer complex destabilization in acidic medium. By changing the pH from 7.4 to 4.0, 5-FU (pKa 8.0) is protonated, causing the cleavage of Cu(II)-5-FU coordinate bond followed by nearly 20% rise in the drug delivery rate. The pH change exerted a greater influence on the DOX in vitro release. No more than 10% of the loaded drug was delivered at pH 7.2 or 6.0 within one week, while at pH 5.0 around 60% of loaded DOX was released, in a sustained manner, over the same period of time. The pH-responsiveness of the DOX-imprinted hydrogel derives from the competitive binding the Lewis acids, protons, and cupric ions to the functional monomer, 4-VPy, acting as Lewis base. At pH 7.2, 4-VPy is fully deprotonated (pKa = 5.2) and strongly chelates the Cu(II) ion. By lowering the pH, the protonated form of 4-VPy is increasing, resulting in the cleavage of the coordinate bond between 4-VPy and Cu(II). Moreover, the DOX-Cu(II) complex is also destabilized at low pH values, facilitating DOX release.

The key elements in the structure of conventional pH-sensitive polymers are different ionizable weak acidic or basic groups, attached to the hydrophobic backbone. Polymers change their properties, such as their swelling degree, as a result of the protonation and deprotonation of acidic (e.g., –COOH and –SO_3_H) and basic (e.g., –NH_2_) functional groups in response to pH changes in the tumor environment. As such, the hydrophobic (collapsed) state will switch to hydrophilic (swollen) configuration, due to the electrostatic repulsions of the generated charges (anions or cations) [10]. Therefore, in order to exploit this approach in designing pH-responsive MIPs, polymers bearing basic functionalities on their structure should be employed, which will become protonated in the acidic pH of tumor environment, however currently reported results show modest to moderate success. In one such attempt, a pH sensitive MIP-based DDS was recently developed for 5-FU delivery [71] and was prepared with a basic monomer, 4-VPy. At both pH 5.8 and 7.4, respectively, the release profiles showed an initial burst (of five days) and then a slow release (up to 30 days) of the imprinted drug. Nevertheless, a modest difference in the release rates within 30 days at acidic (90%) compared to physiological (80%) pH were recorded. By replacing 4-VPy with AA, a very similar behavior, with slightly lower release rates, was observed.

As discussed above, acrylic-type monomers are the most frequently employed in molecular imprinting, including for drug delivery applications. The vast majority of acrylic crosslinkers present in most cases several ester bonds in their structure, making them susceptible to hydrolysis at acidic pH. For example, a TRIM-based MIP exhibited a large increase in the release rate of the imprinted PCX [59] when evaluated at pH 5.0 compared to physiological pH. When higher amounts of crosslinker (an 8-fold increase) were employed in the imprinting process, the PCX release dropped by half. This decrease was attributed to a more rigid polymer network and well-defined imprinted sites which led to a lower release kinetics.

Similarly, PCX-imprinted NPs were synthesized via non-covalent imprinting, in which the carboxylic acid groups of MAA were used to create hydrogen bonds with the functional groups of the template [57]. The acid-catalyzed disruption of hydrogen bonds between the template and functional monomer within the imprinted sites of MIP-DDS may lead to a pH-triggered drug release. An insignificant PCX release under physiological pH (around 1% of the loaded drug) lead to a gradually accelerating release in acidic environment (pH = 6.0 about 7%, while at pH = 5.0 ~15%) during 100 h.

Further attempts to improve drug release of pH-sensitive PCX-imprinted MIPs by increasing polymer porosity was tested via copolymerization of polyhedral 1–3 nm sized oligomeric silsesquioxanes (POSS) nanocomposites [58]. A slow and minimal (3% of the loaded drug) release at pH = 7.0 was recorded after more than 100 h. The release rates of PCX somewhat increased by adjusting the pH to 6.0 and 5.0, delivering 10% and 12%, respectively over the same time period.

Similar attempts of pH triggered release (pH = 5.0) were tested for AZT reporting in this case a three-phased release profile: (i) An initial burst effect with a steep slope, caused by the surface adsorbed AZT molecules, (ii) a medium slope zone corresponding to the entrapped template molecules inside the imprinted cavities that are in the immediate vicinity of the NP’s surface and (iii) a mild slope due to the deeply entrapped AZT molecules within the NP core [69]. In contrast, the NIP NPs showed a burst release, discarding its payload in less than 50 h, because of the non-specific binding of AZT. In case of the imprinted NPs the total drug release was close to 100% after 200 h of exposure.

Another example of pH triggered weakening of template-functional monomer interactions (H-bonds) is a DOX-imprinted MIP-DDS using N-isopropylacrylamide as functional monomer, demonstrating slightly higher drug release efficiency at pH = 5.8 (94%) compared to physiological values (83%).

Nevertheless, up to date a minor success has been achieved in endowing pH responsive drug release of the resulting MIP-DDS by using the acrylic functional monomers most commonly employed in analytical applications. It seems that efforts in optimizing molar ratios of different components of the polymerization formulation for ideal pH responsiveness of the imprinted polymer fade against selecting the appropriate constituent, with the right functionalities, are able to endow the expected mechanical (swelling) or chemical (hydrolytic erosion) transformations triggered by changes in acidity of the environment.

**Figure 5:**
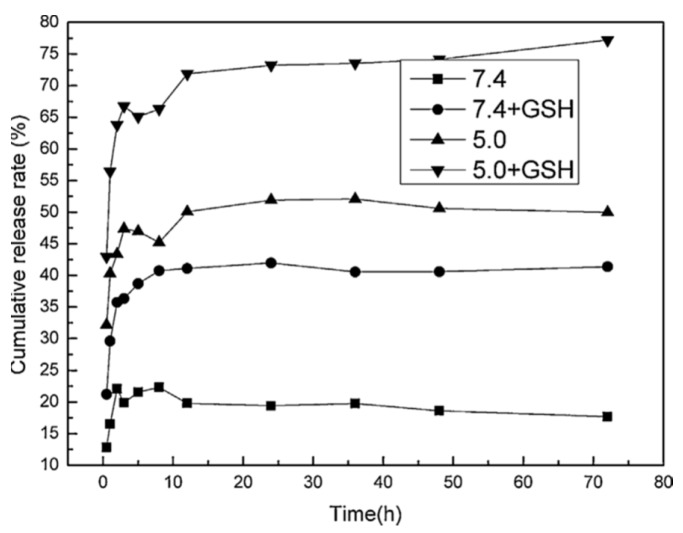
Release curves of doxorubicin from the dual stimuli responsive nanocarrier. Reproduced with permission from [85].

A redox and pH dual-triggered drug delivery based on a disulfide bond containing monomer, namely 2-methacrylester hydroxyethyl disulfide (MABHD), was developed for the delivery of DOX (Figure 5) [85]. As compared to normal cells, cancer cells present a higher concentration of glutathione (GSH), which would cleave the S–S bonds thus triggering the DOX release. The obtained DOX-imprinted NPs were tested in acidic and physiological media (pH 5.0 and 7.4, respectively), in the absence and presence of GSH. The least amount of released DOX, of about 22%, was observed at pH = 7.4, in the first 10 h, and a slight re-adsorption was seen within the next hours, with no obvious subsequent release. When 10 mM GSH was added over the MIP NPs, at physiological pH, the drug release rate increased, due to the cleavage of the S–S bonds in the polymer, achieving a 42% release of DOX after 24 h. The percent drug release raised to 50% under acidic conditions (pH = 5.0), the process being facilitated by the disruption of the hydrogen bonds between the drug and polymer. In the presence of both stimuli, acidic pH and GSH, DOX release increased up to 70%. Because a similar effect on the release process was observed for both GSH and pH 7.4, it seems that pH changes do not affect the S–S bonds. Thus, dual triggered drug delivery could be an efficient and safer way of delivering antitumor agents inside the tumor cells, with less damage to normal cells.

Recently, Liu et al. [76] developed a redox-mediated degradable nanocarrier capable of releasing nitric oxide (NO) in a controlled manner; the release being triggered by the high GSH concentrations in cancer cells (Figure 3). Once the S-nitrosothiols containing hollow double-layer imprinted NPs infiltrates into the cancerous cells, the disulfide bridge of N-bis(acryloyl)cysteamine on the outer polymeric shell is cleaved by the high amounts of GSH, triggering the degradation of the second layer and subsequently by the trans-nitrosation mechanism NO is released. Adding 5 mM of GSH in pH = 7.4 buffer, the S-nitrosothiols NPs released in a short amount of time its payload. Simulating the normal conditions present in the healthy human tissues (3 μM GSH) an inevitable, but physiologically insignificant leakage of NO (t_1/2_ of 445 min and a T_[NO]_ of 0.3 μmol·g^−1^) is observed, while at elevated GSH levels (5 mM) an immediate release of the payload (t_1/2_ of 145 min and a T_[NO]_ of 1.7 μmol·g^−1^) is recorded.

Magnetic NPs play a very important role in smart delivery. A triggered drug release occurs at the targeted area under the influence of an external magnetic field. By combining the magnetic properties of magnetite NPs with MIP technology, efficient drug nanocarriers responsive to external stimuli can be developed, avoiding the bulk heating of the surrounding tissue.

Even though many papers report the preparation of core-shell magnetic MIP NPs-based DDS for cancer treatment, only several of them evaluated the influence of the external magnetic field on the drug release rate.

For example, Kazemi et al. [86] synthesized magnetic MIP NPs (~100 nm) for letrozole via non-covalent imprinting, using MAA as functional monomer, TRIM as cross-linker, and MAA-modified magnetite as magnetic core. Under the influence of an external alternative magnetic field (AMF), the weak hydrogen bonds are disrupted, leading to an increase in the drug release. Exposing the MIP for 240 min to a magnetic field of 150 G, at pH = 7.4, the release rate increased from 33% (no AMF) to 55%. Doubling the AMF strength, a further increase could be observed, up to 61%. A similar behavior under AMF was observed for the NIP release rate: 15%, 41%, and 46%, respectively.

**Figure 6:**
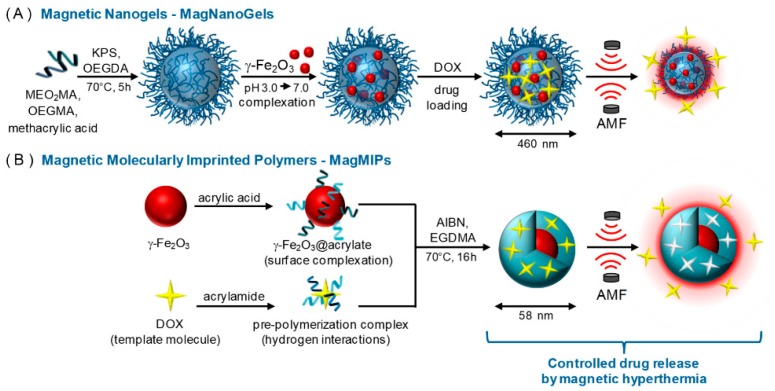
Schematic illustration of the synthesis of (**A**) MagNanoGels by precipitation radical copolymerization and post-assembly of magnetic nanoparticles (MNPs) inside nanogels and (**B**) MagMIPs via a subsequent grafting of an acrylic acid compound on the surface of MNPs and the growth of the polymer in the presence of DOX for imprinting polymerization. Loading and release of DOX under an alternative magnetic field. Reproduced with permission from [87].

Two magnetic nanosystems for DOX controlled release were developed and tested in vitro and on human prostatic cancer cells (PC-3 cancer cells) under AMF excitation. The first one was represented by magnetic nanogels made of thermosensitive and biocompatible polymers (Figure 6A), loaded with the antitumoral drug, and the second one was based on magnetic core DOX-imprinted polymeric shell NPs (Figure 6B) [87]. In this case, both magnetic nanogels and NPs act as individual “hot spots” to generate localized heating that triggers the release of DOX without raising the global temperature. Upon 4 h exposure to AMF, the increase in DOX release, from 24% to 45% of the total payload, was attributed to conformational changes in the nanogel’s polymer network, whereas for the six-fold increase (from 10% to 60%) in case of MIP NPs the magnetic field driven disruption of the hydrogen bonds between the drug and the polymer is accounted for. However, the released amount of DOX from the nanogel in 4 h is much higher (16.7 μM) compared to MIP (7 μM), proving the specific binding of DOX in the imprinted cavities in contrast to the DOX-loaded nanogel. In the presence of AMF a decrease in the cell viability of the PC-3 cancer cells exposed to the both DDS was recorded (from 54% to 30% for the DOX-loaded nanogel and from 88% to 60% in the case of the MIP NPs, respectively).

**Figure 7:**
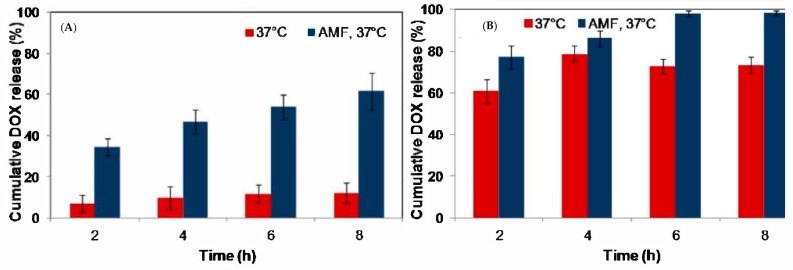
Cumulative DOX release in percent versus time of Fe_2_O_3_@DOX-MIP (**A**) and the non-imprinted magnetic core shell type nanoparticles (Fe_2_O_3_@NIP-DOX NPs) (**B**) ((Fe) = 50 mM) at 37 °C without magnetic field (red) and under AMF (335 kHz, 9 mT, blue). Partially reproduced with permission from [68].

The combination of the sustained drug release capability of MIPs and the magnetic properties of iron oxide NPs was exploited in another study for the controlled release of DOX [68]. The applied AMF was able to produce an increase in the release rate of the drug from the synthesized DOX-imprinted magnetic core-shell type NPs (Figure 7). The in vitro release studies performed at 37 °C, showed that MIP magnetic NPs exposed to AMF for 8 h, eliminated 60% of the loaded drug, four times more than the same polymer in the absence of the external stimulus. The corresponding NIP NPs, evaluated under the same experimental conditions, released completely its load after 8 h (98%). However, DOX release was also high when no AMF was applied, due to the non-specific binding (physical adsorption) of the drug within the non-imprinted polymer. The potential applicability of the magnetic MIP was evaluated on PC-3 cancer cells, in the presence and absence of AMF. After 90 min only a 10% reduction of the cell viability was induced by the MIP-DDS, however in the presence of AMF, cell viability decreased to 60% due to the accelerated DOX release, without any significant temperature elevation of the medium.

The magnetic NPs exposure to an external AFM may induce a local magnetic hyperthermia which could determine the phase transition of a temperature responsive polymer present on NP’s surface and achieve controlled release by magnetism regulation. One popular thermoresponsive polymer is poly(N-isopropylacrylamide) (PNIPAM), extensively explored for drug delivery applications because of its lower critical solution temperature (LCST) of 32 °C, which is well below normal body temperature. By adjusting the temperature, a reversible phase change process takes place, from hydrophilic to hydrophobic. The hydrophilic state, in which drug loading is achieved, is characterized by expanded and flexible polymeric chains and appears at temperatures below LCST. Above LCST, PNIPAM forms globules, the transition to the hydrophobic state being associated with the disruption of the drug-polymer interactions.

The combination of PNIPAM-based thermoresponsive polymers with Fe_3_O_4_ magnetic NPs with superparamagnetic properties was employed in the imprinting of two antitumoral drugs, namely 5-FU [88] and CUR [89], in order to modulate their release by external temperature control (no AMF). The lowest released amount of 5-FU was observed at 25 °C, where nearly 70% of the loaded drug was released within 100 min, reaching 75% at 35 °C. The highest percentage of 90.75% was attained at 45 °C, at which temperature polymer shrinkage and transition towards the hydrophobic state occurs, leading to the disruption of the hydrogen bonds between the template and the polymer’s functional groups.

The ability of CUR-imprinted NPs to release the target molecules can also be adjusted by external temperature. At 25 °C, below LCST of NIPAM, the initial CUR burst release reaches 45% in the first 7 h. By raising the temperature to 38 °C, above the LCST of the monomer, the percentage of CUR released attained 86%. Therefore, temperature can play an important role in modulating drug release rate by determining physio-chemical changes in the polymer.

Drug delivery triggered by an external stimulus may provide temporal control and modulate the amount of drug released, thus improving therapeutic efficiency and reducing systemic toxicity. A different external stimulus was used by Bakhshizadeh et al. [90] to generate free hydroxyl radicals to kill cancer cells. When exposed to X-ray radiation, some materials such as TiO_2_ act as a scintillator and display luminescence properties which activate photosensitizing molecules into producing free radicals. The molecular imprinting technique was used to design hybrid nanodevices containing TiO_2_ NPs and mitoxantrone, a photosensitizer whose absorption spectrum approximately matches the emission spectrum of TiO_2_ NPs.

## 6. Active Targeting

One particular type of controlled DDS is represented by the targeted delivery systems, which are able to provide spatial control of drug release to a specific site of action in the body. This is especially important in cancer chemotherapy, where cytotoxic drugs cause severe damage not only to cancerous cells, but also to normal cells. Designing DDS that are able to recognize senescent cells [91], cancerous cells, and to deliver the load in a controlled manner inside these cells, would reduce the severe side effects usually associated with cancer treatment and improve the therapeutic index of antitumoral agents.

The induction of an external magnetic field to conduct the magnetic drug carrier loaded with the anticancer agent, can be an effective way of tumor-targeting as demonstrated by Asadi et al. in a recent study on a rat animal model [92]. Magnetic-guided drug delivery used magnetite (Fe_3_O_4_) NPs (superparamagnetic iron oxide NPs or SPIONs) coated with a 5-FU-imprinted fluorescent shell for directing the nanocarrier towards the liver. After 24 h following the injection of the magnetic NPs, these were concentrated in the liver area where the magnetic field was present, whereas when no external guidance was used, the NPs were distributed all over the body.

Active targeting of NPs in order to deliver drugs to the affected sites can be achieved by taking advantage of the differences between normal cells and cancer cells. The NPs can be modulated to selectively identify the cancerous cells by detecting the aberrant cancer specific markers, such as over-expressed proteins, enzymes, glycans, etc.

For example, hollow double-layer sialic acid-imprinted polymer NPs containing S-nitrosothiols were developed by Liu et al. [76] for tumor-specific release of nitric oxide (NO). The synthesized MIP was able to selectively bind to cancer cells featuring high levels of sialic acid at the imprinted sites via the formation of stable boronate esters between the phenylboronic acid functionalities of the MIP and the vicinal diol groups of the sialic acid. The selective recognition of the imprinted polymer was demonstrated in vitro on several cancer cell lines featuring different expression levels of SA glycans. The MIP NPs specifically preferred the SA over-expressed HepG2 cells and proved the cell endocytosis capabilities of the polymer. Furthermore, the specific binding of the imprinted NPs was also evaluated in vivo, on HepG2-bearing mice. The MIP NPs were distributed to a much greater extent around the tumor site and also in higher amount in liver, compared to the non-imprinted NPs.

Considering that human epidermal growth factor receptors (such as HER2) are over expressed in several types of ovarian cancers, NPs bearing specific ligands capable of recognizing these receptors may be used to target ovarian carcinoma. For instance, Hashemi-Moghaddam et al. [93] synthesized a MIP-based artificial receptor for HER2 protein using a conformational epitope of HER2 as template and dopamine as monomer, on the surface of silica NPs. Along with this template, DOX, as antitumoral payload, was also added in the polymerization mixture, achieving a DOX-epitope-double imprinted polymer. The obtained NPs were tested in vivo, on human ovarian tumor-bearing mice. Results showed that the DOX concentration in tumor was higher in the group treated with DOX-epitope-MIP NPs, compared to all other groups (DOX-MIP, epitope-MIP, DOX, and control group) and DOX concentration in all other organs was lower in case of double imprinted NPs. Moreover, the tumor volume was also significantly smaller for DOX-epitope-MIP group, greatly enhancing the mice life span.

**Figure 8:**
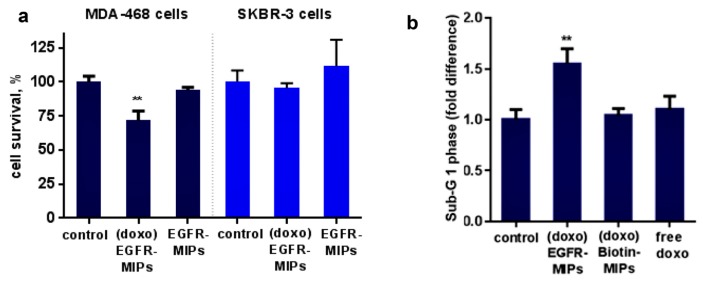
Toxicity assays. (**a**) MTS test performed on MDA-MB-468 and SKBR-3 cells treated with EGFR-nanoMIPs either loaded with doxorubicin (doxo-EGFR-MIPs) or unloaded (EGFR-MIPs). (**b**) Increase of the level of MDA-MB-468 cells in the sub-G1 phase due to the binding of doxo-EGFR-nanoMIP and doxo biotin-nanoMIPs to cells and free doxorubicin (at 100 nM concentration) analyzed by fluorescence-activated cell sorting (FACS). The control represents cells incubated in the absence of nanoMIPs. Reproduced with permission from [94]. Double asterisks indicate P < 0.01.

In another study, Piletsky et al. [94] developed a double-imprinted nanoMIP for DOX and a linear epitope of a tyrosine kinase receptor, namely epidermal growth factor receptor (EGFR), over-expressed in many tumors. Testing nanoMIPs on different cancer cell lines, which expressed no or high amounts of EGFR, the double-imprinted polymer exhibited cytotoxicity, and apoptosis only in those cells that over-expressed EGFR (Figure 8). The control polymer, imprinted with DOX and biotin, did not show any specific binding to the protein over-expressed cells.

## 7. Biocompatibility and Biodegradability of MIP-DDS

Acrylic monomers, such as MAA, MMA, Am, 4-VPy (as functional monomers), and EDMA, TRIM, MBA (as crosslinkers), have been used and continue to be used in the design and development of DDS, and in numerous studies they are presented as holding non-toxic and biocompatible properties. Additional research is however needed in order to evaluate their long-term toxicity and biocompatibility, and most importantly, their biotransformation inside the human body. The polymeric matrix carrying the antitumor drug ideally should be broken down inside the body into non-toxic natural waste products such as water and CO_2,_ through chemical or enzyme-catalyzed hydrolysis. Without doubt, one of the safest ways of synthesizing DDS for biomedical applications is by employing naturally occurring biodegradable polymers, such as protein-based polymers (e.g., albumin, gelatin, collagen) and polysaccharides (e.g., chitosan, dextran, alginate, hyaluronic acid, cyclodextrins).

Gelatin and dextran were used to prepare biocompatible and biodegradable imprinted-like biopolymeric micelles (IBMs) for CUR delivery. First, gelatin-dextran conjugates were synthesized by Maillard reaction, followed by crosslinking gelatin with genipin. Tea polyphenol was used as dummy template, due to its low cost. Template-polymer interactions were based on hydrogen bonding and hydrophobic interaction between hydrophobic proline residues of gelatin and the phenol groups of the dummy template. The IBMs featured a loosely fixed structure, with core-shell micellar constructs and a size of about 200 nm, and were able to preferentially bind polyphenol drugs, such CUR, over the non-analogous polyphenol drugs. Using IBMs, CUR release is retarded, around 50% of the encapsulated CUR being released after 72 h [95]. The cytotoxicity studies performed on HeLa cancer cells did not bring evidence, however, of a notable superiority of the CUR-loaded IBMs as compared to free CUR.

**Figure 9:**
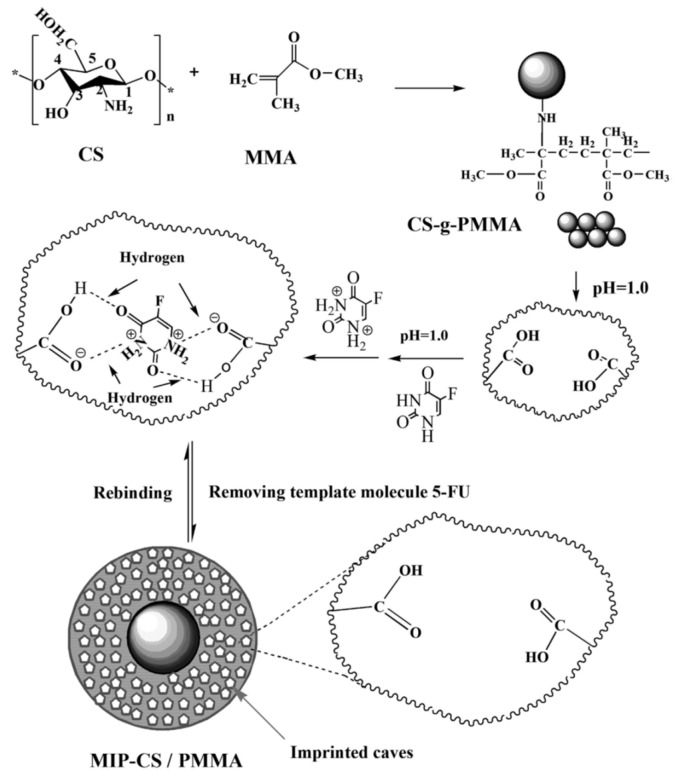
Schematic expression of chemical reaction process to prepare 5-FU imprinted microspheres MIP-chitosan(CS)-g-poly-methyl-methacrylate (PMMA). Reproduced with permission from [96].

One of the most widely used polysaccharide-based polymer for drug delivery applications is chitosan—produced by deacetylation of chitin. The presence of amino and hydroxyl functional groups on its polysaccharide chain provides sites for template interaction and for grafting of additional functionalities. For example, Zheng et al. [96] synthesized 5-FU-imprinted microparticles by grafting methyl methacrylate (MMA) on the surface of chitosan particles (Figure 9). The subsequent hydrolysis gives rise to carboxylic groups able to ionic bonds with the protonated nitrogen atom of 5-FU. The study does not evaluate, however, the effect of the modification of the biopolymer on the toxicity of the resulting MIP. Despite the use of a biodegradable backbone, additional studies assessing the long-term toxicity and biodegradability should be performed when a copolymer is prepared with a non-degradable monomer.

In a similar manner, tannic acid, a biodegradable polyphenol, is modified with methacryloyl chloride in order to introduce crosslinking methacrylic groups [92]. The resulting crosslinker agent was used to prepare a 5-FU-imprinted MIP shell onto SiO_2_ magnetic NPs. Tannic acid is fully biodegradable and presents multiple hydroxyl and carboxyl groups capable of interacting with a drug template molecule. Upon modification with methacrylic groups, its biodegradability needs to be re-evaluated. Degradation studies of the prepared MIP were realized at different pH values: pH = 11, similar to kidney and intestinal environment, pH = 3, mimicking the stomach environment, and physiological pH (7.4). The fastest degradation rate was recorded at extreme pH values (pH = 3 and pH = 11), however, within 12 days, more than 80% of polymer still remained unchanged. The relative cytotoxicity of the MIP NPs was assessed during a seven day incubation timeframe on NIH/3T3 cell-line, and a 10% drop of the cell viability was observed. For biocompatibility qualitative evaluation, the cell viability test was performed on human embryonic kidney (HEK293) cells, over a period of three days. No changes in the morphology or cell death was observed. Nevertheless, further biodegradability and biocompatibility evaluation of the modified natural polymers is required, for longer period of times, and on in vivo animal models.

One of the widely used degradable aliphatic polyester used for drug delivery applications, and especially in long-term implantable devices, is poly(caprolactone) (PCL) [97]. Under physiological conditions it is very slowly degraded (several years for pure PCL) by hydrolysis of its ester bonds [10]. PCL was approved by FDA for different biomedical applications, drug delivery included. In order for PCL to be suitable for the design of molecularly imprinted DDS, its reactivity needs to be improved by introducing new functionalities able to interact with template molecules. For example, mitoxantrone, a photosensitizer, was imprinted employing MAA as functional monomer and diacrylated-modified PCL as crosslinker, in a mixture of DMSO-chloroform [90]. The radical polymerization was achieved on the surface of TiO_2_ NPs, and the optical and radio properties of the obtained MIP-based particles were evaluated on two cancer cell lines. Following X-ray treatment of MIPs, the loaded-mitoxantrone absorbed the emitted photons released from the TiO_2_ NPs and generated toxic hydroxyl radicals. However, the cytotoxic effects on normal human cells were not investigated.

A different polymer with proven biodegradability and no long-term toxicity in rat models [98] is polydopamine (PDA). It is obtained by autoxidation of dopamine, a neurotransmitter involved in many physiological processes [99]. The self-polymerization of dopamine is a straightforward process, frequently used to generate an adherent PDA coating on different particles (e.g., AuNPs [100], magnetic NPs [101], polymeric NPs [102]). Hashemi-Moghaddam et al. employed PDA in MIP development for an antitumor drug release. DOX [93,103] and 5-FU [64] PDA-based imprinted polymers were used to coat magnetic and silica NPs. The imprinted polymers showed a fast release of DOX and 5-FU, as 80% of template drug was released from the NPs within 4 h, with a maximum release after 24 h [64,93,103]. One important advantage of MIP-based PDA preparation is however, the use of mild alkaline aqueous media, which allows the synthesis of water-compatible MIPs.

Another practical approach for developing biocompatible and water-compatible MIPs is the use of hydrophilic monomers, such as HEMA. The MMC-imprinted magnetic NPs [104], prepared with HEMA, were highly dispersive in water due to hydroxyl groups on the surface of NPs, and the template release followed a swelling-controlled diffusion mechanism. In another study [55], HEMA was used to increase the hydrophilicity of the PCX-imprinted microparticles. As a result, the polymer was more sensitive to changes in the pH of the aqueous medium, which upon a swelling effect at higher pH values (7.4) facilitates PCX release. An important increase in the swelling ratio of HEMA-containing particles with pH was also reported for 5-FU imprinting [51]. Furthermore, HEMA was used in the synthesis of imprinted hydrogels for sustained released of 5-FU [52,105].

Unfortunately, most imprinted polymers for DDS applications are prepared in aprotic and low polarity organic solvents in order to favor and conserve the hydrogen bond interactions between the template and the functional monomer. The most common solvents used for MIP synthesis are toluene, chloroform, dichloromethane, and ACN. Moreover, porogenic solvents play an important role in the formation of the polymeric porous structure, influencing polymer morphology and directly affecting the MIP performance. However, with respect to drug delivery, the presence of residual organic solvents can induce cellular damage. Moreover, drug delivery applications require MIPs capable of working in aqueous media. Therefore, a shift toward MIP synthesis in aqueous solutions should be produced, by employing hydrophobic, ionic, or metal co-ordination interactions to enhance template-functional monomer associations in water [106].

If the polymeric material employed is not biodegradable, the features of the polymeric drug-carrier should be optimized to allow its clearance from the body after drug release. Unfortunately, most studies neglect to follow the postdelivery fate of the polymers, although the majority of the MIP-based DDS for cancer treatment are derived from acrylic monomers [10].

Magnetic NPs are frequently used for DDS development intended for cancer treatment, because they can be easily tracked, manipulated, and directed towards tumor site using external magnetic field. Iron oxide NPs are the only approved types of magnetic NPs for biomedical applications [107], magnetite being one of the naturally occurring iron oxides in human heart, liver, and spleen [103]. However, for in vivo applications their surface needs to be coated with biocompatible materials, since it was shown that they can induce severe toxicity via protein denaturation [108]. PDA-coated magnetic NPs were synthesized for DOX [103] and 5-FU [64] imprinting. The resulting NPs showed good biocompatibility and their aggregation and oxidation was inhibited by the PDA-based MIP coating.

## 8. Disambiguation of Modified-Release DDS Types

DDS can control the mechanism of drug release, i.e., the rate and/or the location of drug release. From this point of view, DDS can be classified into two major groups: Immediate-release and modified-release dosage forms. The latter group can be further divided into delayed-, extended-, and targeted-release systems. Sustained and controlled release systems are both subtypes of the extended-release dosage forms. While in the case of the immediate release, the drug is released shortly after administration, the modified release ensures that the drug release occurs either at some point after the initial administration (delayed release), or over a prolonged period of time (extended-release), or to a specific biological target (targeted-release). Both extended-release dosage forms (i.e., sustained-release and controlled release forms) are able to maintain the rate of drug release over a sustained period, but, in addition, controlled-release systems are designed to lead to constant plasma concentrations independently of the biological environment at the administration site. In other words, controlled-release systems regulate not only the release profile of the drug, but also the drug concentration achieved within the body [109].

Although various forms of drug release (i.e., immediate release, modified release, delayed release, and extended release) are clearly characterized by the FDA and distinct pharmacopoeias, no strict definition is provided for the controlled and targeted release. As such, usually, the aforementioned terms in the literature covering the use of MIPs as DDS are confusingly misused.

## 9. In Vivo Evaluated Imprinted DDS

### 9.1. Cancer Therapy

The vast majority of MIP-based DDS intended for cancer therapy (Table 2) are still at an early stage of the preclinical evaluation, that is material characterization and in vitro drug release studies.

A small number of formulations imprinted with 5-FU and DOX have been advanced to in vivo testing on animal models. The results unequivocally demonstrated that by loading the chemotherapeutics within MIP-based DDS, led to a better control of tumor growth, to an increase in survival rates, and to a reduction of the side effects in comparison with conventional therapeutic protocols [64,67,93].

5-FU imprinted NPs (polymerization of AM and EDMA) were evaluated by Gardouh et al. [67] for the ability to arrest cancer cell growth, induce apoptosis, and restrain angiogenic responses in an Earlich ascites carcinoma xenograft model. The imprinted nanoformulation showed higher antitumor effect as compared to the free 5-FU as indicated by the enhanced apoptosis and the reduction in tumor weight. Moreover, the decreased liver toxicity was correlated with the preferential localization of the chemotherapeutic at the tumor site. In a study reported by Hashemi-Moghaddam et al. [64] magnetic NPs coated with 5-FU imprinted PDA were evaluated for the controlled delivery of the chemotherapeutic in a breast adenocarcinoma mice model. The drug uptake controlled by an external magnetic field resulted in a higher efficacy in suppressing tumor growth, increasing survival rate, and reduction of side effects for the 5-FU imprinted polymer. Molecular imprinting was used by Hashemi-Moghaddam et al. [93] to design an artificial tumor specific antigen, i.e., an artificial receptor for the HER2 protein. The group reported the design of a targeted DDS based on molecularly imprinted PDA, where both the epitope of the HER2 protein and the antitumor drug DOX were used as templates. The carrier was evaluated for the targeted delivery of DOX in an ovarian cancer xenograft model and results indicated the successful targeting of the chemotherapeutic, followed by an improved control of tumor growth and increased survival rates.

MIP-based formulations of molecules known for their low solubility, poor absorption, rapid degradation, or excretion—CAP and CUR—were evaluated in vivo on animal models and results showed an increase in the bioavailability of the drugs as compared to the respective free compounds [54,95]. The increase of the bioavailability of CAP, a prodrug of 5-FU, was reported by Mo et al. [54] for a floating oral DDS. The cooperative effect of MAA as monomer, and oligomeric liquid crystalline silesquioxanes as co-monomers, was exploited for the successful imprinting of CAP to design a carrier with good floating properties and sustained release of the template. Zhang et al. [95] reported an imprinted-like biopolymeric micelle for CUR delivery fabricated via the co-assembly of gelatin- dextran conjugates and tea polyphenol. The carrier enhances the solubility of the drug and, as a consequence, significantly improves its bioavailability after oral administration.

### 9.2. Other Therapeutic Applications

The sustained and stimuli-responsive release of bioactive compounds has been explored in the development of MIPs as delivery systems for various administration routes (e.g., ocular, dermal, oral, or intravenous) of other therapeutics as well [12,13]. Examples include but are not limited to antipyretics [112], anti-Alzheimer drugs [113] and antipsychotics [114] smoking cessation agents [115], and β-adrenergic antagonists [116].

To date only a few drug-imprinted devices have been evaluated in vivo for the delivery of drugs. As examples, for the timolol maleate imprinted soft lens (polymerization of MAA, *N,N*-diethylacrylamide, and EDMA) [117], the ketotifen fumarate imprinted ophthalmic device (polymerization of acrylic acid, acrylamide, *N*-vinyl 2-pyrrolidone, PEG 200 dimethacrylate, and HEMA) [118] and the ciprofloxacin imprinted soft lens (polymerization of acrylic acid, HEMA, 3-(tris(trimethylsiloxy)silil)propyl methacrylate, polyvinylpyrrolidone, and EDMA) [119] the results of the tests on animal models showed that the MIP carriers are able to circumvent the main issues usually linked to conventional formulations for ophthalmic use, i.e., poor bioavailability as a result of the short residence time and the lack of sustained release of the bioactive at high titers and in long duration [120]. Indeed, apart from the excellent biocompatibility, the formulations presented extended release time and prolonged permanence time of the bioactive as compared to the respective commercially available eye drops.

## 10. Conclusions and Future Perspectives

Conventional primary or adjuvant chemotherapy is associated with high non-specific toxicity and severe side-effects, due to the poor pharmacodynamic selectivity and unfavorable pharmacokinetic profile of the current anticancer drugs. However, the use of smart polymeric (nano)carriers play a significant role in improving treatment efficiency through localized, temporized, and dose-controlled delivery of the antitumor agents, especially when guided by endogenous or exogenous stimuli. As one step further, by incorporating the chemotherapeutics into the polymeric matrix by molecular imprinting not only a significant increase of the loading capacity, but an extra boost in the tunability of the drug release kinetics and stimuli responsiveness of these carriers may be achieved. Despite the advances made in the synthesis of molecularly imprinted polymers intended for drug delivery, the development of MIP-DDS for cancer therapy is still in its infancy. Unfortunately, to this date, the majority of studies dealing with MIP-based drug delivery systems employ formulations initially tested for analytical applications. It appears that little effort is channeled towards adapting the MIP formulations to the specific needs of drug delivery in line with the current pharmaceutical and biomedical regulations, and considering minimal safety profile requirements. Moreover, due to the limited translatability of MIP-DDS performance and cellular toxicity recorded on simple in vitro models, a significant increase of studies progressing to in vivo testing on animal models is more than desired.

Therefore, a great deal of research is yet to be undertaken on MIP-DDS development to overcome some of the issues related to the imprinting process currently not complying with the modern pharmacotherapeutic requirements, to assess their safety profile, and finally to reach the ultimate goal, clinical translation.

## Figures and Tables

**Table 1:** Particularities of the molecular imprinting process for analytical purposes and drug delivery.

Choices in Molecular Imprinting/Expected Features	MIPs in Analytical Sciences	MIPs as DDS
Imprinting technique (template/monomer interaction)	VARIABLE non-covalent>>covalent, pivot based	VARIABLE non-covalent>>pivot based
Monomer selection	NO CONSTRAINS	LIMITATIONS Biocompatible, biodegradable, Particular functionalities for stimuli-responsive MIPs
Cross-linker selection	NO CONSTRAINS	LIMITATIONS Biocompatible and biodegradable with impact on MIP performance
Polymerization initiation/MIP morphology	NO CONSTRAINS Photo-, thermal initiation, electropolymerization/Bulk polymer; NPs; films	CONSTRAINS Photo-, thermal initiation/Bulk polymer (hydrogels); NPs
Template removal	CRITICAL sometimes tedious	NO NEED
Well-defined, homogenous binding sites	HIGH High IF for specific re-binding—improved selectivity	HIGH/VARIABLE Improved drug loading/Combined release profiles
Degree of cross-linking	HIGH Polymer rigidity affecting selectivity	VARIABLE Adjustable release kinetics
Solvent (porogen)	VARIABLE (aprotic favored in non-covalent imprinting) Porosity important	IDEALLY WATER Traces of non-aqueous solvents may be toxic Porosity affecting release kinetics

**Table 2:** MIP-based drug delivery systems (DDSs) intended for cancer therapy.

Active Drug (T)	Imprinting Approach Polymerization Mixture (M/C/I/S)	DDS Type/Targeted Delivery	Release Mechanism Drug Load; Drug Release	Biocompatibility/Biodegradability	Development Stage	Ref.
5-Fluorouracil	Cu(II) mediated imprinting MAH, HEMA/MBA/APS, TEMED/water	Implantable cryogel discs/-	Swelling-controlled drug release Drug release—80%/9 h (pH 7.4, 37 °C)	Yes/No (acrylic Ms, high Cu(II) load)	Material characterization In vitro release studies	[78]
5-Fluorouracil	Non-covalent imprinting AM/EDMA/AIBN/ACN + methanol	Nanospheres/-	Release mechanism—diffusion/erosion Drug release—60%/10 h (pH 1.2–6.8, 37 °C)	Yes/No (acrylic Ms)	Material characterization In vitro release studies In vivo studies: Female Swiss albino mice inoculated with human Earlich ascites carcinoma cells xenograft	[67]
5-Fluorouracil	Non-covalent imprinting Dopamine/water	MIP-coated Fe_3_O_4_ NPs/Magnetically assisted DD	Release mechanism—diffusion/erosion Drug release—80%/4 h (pH 7.4, 37 °C)	Yes/Yes	Material characterization In vitro release, cytotoxicity studies In vivo studies: Breast adenocarcinoma in Balb/c mice	[64]
5-Fluorouracil	Non-covalent imprinting Crosslinked tannic acid/AIBN/hexadecane + SDS in water	MIP-coated Fe_3_O_4_@SiO_2_@FITC-MPS NPs/Magnetically assisted DD	Release mechanism—diffusion/erosion Drug release—70%/80 h (pH 7.4, 37 °C)	Yes/Yes	Material characterization In vitro release, cytotoxicity studies In vivo pharmacokinetic studies: Healthy male Wistar rats	[92]
5-Fluorouracil	Non-covalent imprinting MAA or HEMA/EDMA /AIBN-ACN	Microparticles with polymer functional brushes (FB) (PMAA, PHEMA, PNIPA)/-	Stimuli responsive drug release (pH—MIP with PMAA FB; temperature—MIP with PNIPA FB) Drug load—32.3 μmol·g^−1^ for MAA MIP with PMAA FB; 28.8 μmol·g^−1^ for HEMA MIP with PHEMA FB; 27.6 μmol·g^−1^ for MAA MIP with PNIPA FB Drug release—82%/24 h (pH 10) vs. 41%/24 h (pH 2) for MAA MIP with PMAA FB; 96%/24 h (pH 10) vs. 68.5%/24 h (pH 2) vs. for HEMA MIP with PHEMA FB; 50%/24 h (20 °C) vs. 21%/24 h (40 °C) for MAA MIP with PNIPA FB	Yes/No (acrylic Ms)	Material characterization In vitro release studies	[51]
5-Fluorouracil	Non-covalent imprinting Chitosan, MMA/APS/water	Microspheres/-	Stimuli responsive release (pH) Drug load—96 mg/g (pH = 1.2) Drug release—48%/2 h and 95%/30 h (pH 7.4, 20 °C) vs. 2.5%/30 h (pH 1.2, 20 °C), 30% (pH 4, 20 °C), 62% (pH 6.8, 20 °C)	Yes/No (acrylic Ms)	Material characterization In vitro release studies	[96]
5-Fluorouracil	Non-covalent imprinting NIPA/MBA/APS/water	MIP-coated Fe_3_O_4_@C_Si_ nanospheres/Magnetically assisted DD	Stimuli responsive release (temperature); Drug load—96.53 mg/g Drug release—70% (25 °C)/1.6 h, 91.2%/1.6 h (45 °C);	Yes/No (acrylic Ms)	Material characterization In vitro release studies	[88]
5-Fluorouracil	Non-covalent imprinting AA or 4-Vpy/EDMA/AIBN/ACN + methanol	MIP-coated Fe_3_O_4_@SiO_2_@FITC-MPS NPs/Magnetically assisted DD	Stimuli responsive release (pH) Drug release—90%/30 days (pH = 5.8, 37 °C) vs. 70%/30 days (pH = 7.4, 37 °C) for 4-Vpy MIP	Yes/No (acrylic Ms)	Material characterization In vitro release studies	[71]
5-Fluorouracil	Non-covalent imprinting MAA, HEMA/EDMA/AIBN/-	Hydrogel/-	Swelling-controlled drug release Drug load—0.0914 mg/g Drug release—30%/5 h (pH 6.8, 25 °C)	Yes/No (acrylic Ms)	Material characterization In vitro release studies	[52]
5-Fluorouracil	MAA/EDMA/AIBN/ACN	Nanospheres	Mechanism of release—diffusion/erosion Drug load Drug release—40%/10 h (pH 7.4, 37 °C) (burst release); 80%/96 h (pH 7.4, 37 °C)	Yes/No (acrylic Ms)	Material characterization In vitro release, cytotoxicity studies	[53]
5-Fluorouracil	Non-covalent imprinting AA, HEMA/MBA/APS, TEMED/water	Hydrogel/-	Swelling-controlled drug release Drug load—0.875 mg/g Drug release—45%/5 h (37 °C)	Yes/No (acrylic Ms)	Material characterization In vitro release studies	[105]
Capecitabine	Non-covalent imprinting MPDE (LC), POSS, MAA/EDMA/AIBN/toluene + ACN	POSS-LC nanocomposite (floating oral DDS)/-	Mechanism of release—diffusion/erosion Drug load—164.21 mg/g Drug release—80%/12 h	Yes/No (acrylic Ms)	Material characterization In vitro release, cytotoxicity studies In vivo pharmacokinetic studies: Healthy male Wistar rats	[54]
Doxorubicin	Non-covalent imprinting MABHD, EDMA/DMAP, AIBN/ethanol	MIP-coated mesoporous silica NPs/-	Stimuli responsive release (pH, GSH) Drug load—10.5 ± 0.2 wt.% Drug release—72%/12 h (GSH 10 mM, pH 5, 37 °C) vs. 22% (no GSH, pH 7.4, 37 °C) and 42% (GSH, pH 7.4, 37 °C)	Yes/No	Material characterization In vitro release, cellular uptake cytotoxicity studies	[85]
Doxorubicin	Cu(II) mediated imprinting 4-Vpy, HEMA/MBA/APS, SBS/water	Hydrogel	Stimuli responsive release (pH) Drug load—6.74 μmol·g^−1^ Drug release: 10%/7 days (pH 7.2, 37 °C), 60%/7 days (pH 5, 37 °C)	Yes/No (acrylic Ms, high Cu(II) load)	Material characterization In vitro release studies	[80]
Doxorubicin and epitope of HER2 protein (Human epidermal growth factor)	Non-covalent imprinting Dopamine/water	Double imprinted MIP-coated mesoporous silica nanospheres/Targeted delivery of DOX (specific target—HER2)	Mechanism of release—diffusion/erosion Drug load Drug release—most of drug within 4 h, reaching a maximum after 24 h (pH 7.4, 37 °C)	Yes/Yes	Material characterization In vitro release studies In vivo studies: Female C57BL/6 nude mice—SKOV3 human ovarian cancer cells xenograft	[93]
Doxorubicin and epitope of EGFR (Epidermal growth factor receptor)	Non-covalent imprinting NIPA, N-tert-butylacrylamide, AA, N-(3-aminopropyl)methacrylamide/MBA/APS, TEMED/water	Double imprinted nanospheres Targeted delivery of DOX (specific target—EGFR)	Mechanism of release—diffusion/erosion Drug load Drug release	Yes/No (acrylic Ms)	Material characterization In vitro cellular uptake and cytotoxicity studies	[94]
Doxorubicin	Non-covalent imprinting NIPA/EDMA/AIBN/water + ethanol	MIP-coated Fe_3_O_4_ NPs/Magnetically assisted DD	Stimuli responsive release (pH) Drug release—70%/144 h (pH 5.8, 37 °C) vs. 12% (pH 7.4, 37 °C)	Yes/No (acrylic Ms)	Material characterization In vitro release studies	[110]
Doxorubicin	Non-covalent imprinting Dopamine/water	MIP-coated Fe_3_O_4_ NPs/Magnetically assisted DD	Mechanism of release—diffusion/erosion Drug release—90%/8 h (pH 7.4, 37 °C)	Yes/Yes	Material characterization In vitro release studies In vivo studies: BALB/C inbred female mice—papillary breast adenocarcinoma mammary tumor	[103]
Doxorubicin	Non-covalent imprinting AM, AA/EDMA/AIBN/ethanol	MIP-coated Fe_3_O_4_ NPs/Magnetically assisted DD	Alternative magnetic field (AMF)—controlled Drug release Drug load—35.6 μmol·g^−1^ Drug release—60%/8 h (AMF, 37 °C), vs. 12%/8 h (no AMF, 37 °C)	Yes/No (acrylic Ms)	Material characterization In vitro release studies In vitro cellular uptake and intracellular drug release studies	[68,87]
Doxorubicin	Non-covalent imprinting MMA/EDMA/AIBN/water-oil (cetyl alcohol)	MIP doped graphene oxide quantum dots (GQDs) microspheres/-	NIR radiation—controlled release (inductive NIR heating) Drug load—7.08 wt.% Drug release—36.54%/3 h (NIR radiation, pH 7.4) vs. 12%/3 h (NIR radiation, pH 7.4)	No/No (vinylic surfactant, (acrylic Ms)	Material characterization In vitro release studies	[111]
Paclitaxel	Non-covalent imprinting MAA, HEMA/EDMA or TRIM/AIBN/toluene	Microparticles/-	Mechanism of release—diffusion/erosion Drug load—13.32 mg/g (MIP_TRIM_); 9.86 mg/g (MIP_EDMA_) Drug release—85%/50 h (MIP_TRIM_, pH 7.4, 37 °C) and 40%/50 h (MIP_EDMA,_ pH 7.4, 37 °C)	Yes/No (acrylic Ms)	Material characterization In vitro release, cytotoxicity studies	[55]
Paclitaxel	Non-covalent imprinting MPDE (LC), POSS, MAA/EDMA/AIBN/toluene and ACN	POSS-MPDE (LC) nanocomposite/-	Release mechanism—diffusion/erosion Drug load—106.93 μmol·g^−1^ Drug release—rate 4.6 μg/mL/15 h	Yes/No (acrylic Ms)	Material characterization In vitro release, cytotoxicity studies In vivo pharmacokinetic studies: Healthy male Wistar rats	[56]
Paclitaxel	Non-covalent imprinting MAA, MMA/EDMA/AIBN/hexadecane + chloroform and water + SDS	MIP NPs conjugated to PEG-FA (MIP-PEG-FA)/Targeted delivery of paclitaxel (specific target—the folate receptor)	Mechanism of release—diffusion/erosion Drug load—13.1 wt.% Drug release—11.2%/24 h (pH 5, 37 °C), 15%/100 h (pH 5, 37 °C)	Yes/No (acrylic Ms)	Material characterization In vitro release, cytotoxicity studies	[57]
Paclitaxel	Non-covalent imprinting M-POSS, MAA/EDMA /AIBN/ACN	M-POSS microparticles/-	Release mechanism—diffusion/erosion Drug load—17.1 wt.% Drug release—burst release: 10.7%/5 h (pH 5, 37 °C); 12%/100 h (pH 5, 37 °C)	Yes/No (acrylic Ms)	Material characterization In vitro release, cytotoxicity studies	[58]
Paclitaxel	Non-covalent imprinting MAA, MMA/EDMA, TRIM/AIBN/hexadecane, chloroform + water, SDS	NPs/-	Release mechanism—diffusion/erosion Drug load—17.8 wt.% Drug release—37.7%/48 h (pH 5, 37 °C)	Yes/No (acrylic Ms)	Material characterization In vitro release studies	[59]
Curcumin	Non-covalent imprinting (Dummy T: tea polyphenol) Gelatin-dextran conjugates/genipin/water	Polymeric micelles/-	Release mechanism—diffusion/erosion Drug load—100 mg/g Drug release: 54%/72 h (pH 2, 37 °C), 47%/72 h (pH 5, 37 °C), 60%/72 h (pH 6.8, 37 °C)	Yes/Yes	Material characterization In vitro release, cytotoxicity studies In vivo pharmacokinetic studies: Healthy male Sprague–Dawley rats	[95]
Curcumin	Non-covalent imprinting Acryl functionalized β-CD, NIPA/MBA/AIBN/ACN-free radical polymerization	MIP-coated Fe_3_O_4_@SiO_2_@MPS nanocomposite/Magnetically assisted DD	Stimuli responsive release (temperature); Drug load—77 mg/g Drug release—burst release 45%/7 h (25 °C and 38 °C); 62%/3 days (25 °C), 86%/3 days (38 °C)	Yes/No (acrylic Ms)	Material characterization In vitro release studies	[89]
Azidothymidine	Non-covalent imprinting ITC/EDMA/AIBN/ACN	MIP-coated Fe_3_O_4_@SiO2—MPS NPs/Magnetically assisted DD	Stimuli responsive release (pH) Drug load—170.75 mg/g Drug release—burst release 80%/10 h (pH 5, 37 °C), 90%/75 h (pH 5, 37 °C); 15%/75 h (pH 7.4, 37 °C)	Yes/No (acrylic C))	Material characterization In vitro release, cytotoxicity studies	[69]
Sialic acid/S-nitrosothiols	Non-covalent imprinting VPBA, AMMH/BAC, EDMA/BPO/ACN	MIP-coated SiO_2_/P(EDMA-co-bocAmEMA) NPs/Targeted delivery of S-nitrosothiols (thiol mediated cell uptake, specific target—sialic acid over-expressed on cancer cell membrane)	Stimuli responsive release (GSH or Cu(I) triggered release of nitrous oxide, NO) Drug load—2.1 μmol·g^−1^ Drug release—1.8 μmol·mg^−1^, t_1/2_ = 220 min, (200 µM Cu(I), pH 7.4, 37 °C); 1.7 μmol·mg^−1^, t_1/2_ = 145 min, (5 mM GSH, pH 7.4, 37 °C); 0.3 μmol·mg^−1^, t_1/2_ = 445 min, (3 mM GSH, pH 7.4, 37 °C)	Yes/No (acrylic Ms)	Material characterization In vitro release, cytotoxicity studies	[76]
*R-(+)-*thalidomide *S-(-)-*thalidomide *S,R-(±)-*thalidomide	Non-covalent imprinting NVP, MAA, AA/EDMA, TRIM/AIBN/chloroform	Microspheres/-	Release mechanism—diffusion/erosion Drug load Drug release—15%/75 h (*R-(+)-*thalidomide MIP, pH 5.5), 17%/75 h (*S-(-)-*thalidomide and *S,R-(±)-*thalidomide, pH 5.5)	Yes/No (acrylic Ms)	Material characterization In vitro release, cytotoxicity studies	[61]
*R-(+)-*thalidomide	Non-covalent imprinting MAA, 2,6-bis(acrylamido)Py/MBA/AIBN/methanol	MIP-Poloxamer NPs (Physically deposited MIP-Poloxamer 407, or chemically grafted MIP-acrylate-derived Poloxamer)/-	Stimuli responsive release (temperature); Drug load—3.1 μmol·g^−1^ Drug release	Yes/No (acrylic Ms)	Material characterization In vitro release, cytotoxicity studies	[60]
Mitoxantron	Non-covalent imprinting MAA/polycaprolactone diacylate/AIBN/DMSO + chloroform	MIP-coated TiO_2_ NPs/-	Photodynamic effect-based release Drug load Drug release	Yes/No (acrylic Ms)	Material characterization In vitro cytotoxicity studies	[90]
Sunitinib	Non-covalent imprinting MAA/EDMA/AIBN/chloroform	Hydrogel/-	Swelling-controlled drug release Drug load Drug release—58%/6 h; 76%/24 h	Yes/No (acrylic Ms)	Material characterization In vitro release, cytotoxicity studies	[63]
Mitomycin C	Non-covalent imprinting MAH, HEMA/EDMA/KPS/water + PVA	MIP-coated Fe_3_O_4_ NPs/Magnetically assisted DD	Swelling-controlled drug release Drug load—24 μmol·g^−1^ Drug release—90%/5 h (pH 6, 25 °C, mitomycin C load 8 mg/g)	Yes/No (acrylic Ms)	Material characterization In vitro release studies	[104]
Mitomycin C	Cu(II) mediated imprinting MAH, HEMA/MBA/APS, TEMED/phosphate-buffered saline	Implantable cryogel membranes	Swelling-controlled drug release Drug load—8 mg·g^−1^ Drug release—92.5%/5 h (pH 7.4, 37 °C)	Yes/No (acrylic Ms, high Cu(II) load)	Material characterization In vitro release, cytotoxicity studies	[81]
Amygdalin	Non-covalent imprinting 4-Vpy/ EDMA/BPO/ACN	NPs/-	Swelling-controlled drug release Drug load—0.98 mg/g Drug release—50%/24 h (pH 7, 37 °C), 35%/24 h (pH 2, 37 °C)	Yes/No (acrylic Ms)	Material characterization In vitro release, cytotoxicity studies	[72]

T: Template; M: Functional and backbone monomers; C: Crosslinkers; I: Initiators; S: Solvent; *β*-CD: *β*-cyclodextrin; MAH: *N*-methacryloyl-*L*-histidine; AA: Acrylic acid; HEMA: Hydroxyethyl methacrylate; PHEMA: Poly(hydroxyethyl methacrylate); APS: Ammonium persulfate; KPS: Potassium persulfate; TEMED: *N,N,N’,N’*-tetramethylethylenediamine; DMAP: *N,N*-Dimethylaminopyridine; AIBN: Azobisisobutyronitrile; EDMA: Ethylene glycol dimethacrylate; AM: Acrylamide; MBA: *N,N’*-methylenebis(acrylamide); MAA: Methacrylic acid; PMAA: Poly(methacrylic acid); MMA: Methyl methacrylate; TRIM: Trimethylolpropane trimethacrylate; 4-Vpy: 4-Vinyl pyridine; SBS: Sodium bisulfate; SDS: Sodium dodecyl sulfate; POSS: Polyhedral oligomeric silsesquioxanes; M-POSS: Methacryl polyhedral oligomeric silsesquioxanes; MPDE (LC, liquid crystalline): 4-methylphenyl dicyclohexyl ethylene; FITC: Fluorescein isothiocyanate; MPS: Methacryloxypropyl trimethoxysilane; ITC: Itaconic acid; TMPTA: Trimethylolpropane triacrylate; NIPA: *N*-isopropylacrylamide; PNIPA: Poly(*N*-isopropylacrylamide); MABHD: 2-methacrylester hydroxyethyl disulfide; PEG: Polyethylene glycol; FA: Folic acid; GSH: Glutathione; NVP: 1-vinyl-2-pyrrolidinone; BPO: Benzoyl peroxide; ACN: Acetonitrile.
